# Rate variation and estimation of divergence times using strict and relaxed clocks

**DOI:** 10.1186/1471-2148-11-271

**Published:** 2011-09-26

**Authors:** Richard P Brown, Ziheng Yang

**Affiliations:** 1 School of Natural Sciences & Psychology, Liverpool John Moores University, Liverpool L3 3AF, UK; 2 Department of Genetics, Evolution and Environment, University College London, London WC1E 6BT, UK

## Abstract

**Background:**

Understanding causes of biological diversity may be greatly enhanced by knowledge of divergence times. Strict and relaxed clock models are used in Bayesian estimation of divergence times. We examined whether: i) strict clock models are generally more appropriate in shallow phylogenies where rate variation is expected to be low, ii) the likelihood ratio test of the clock (LRT) reliably informs which model is appropriate for dating divergence times. Strict and relaxed models were used to analyse sequences simulated under different levels of rate variation. Published shallow phylogenies (Black bass, Primate-sucking lice, *Podarcis *lizards, Gallotiinae lizards, and Caprinae mammals) were also analysed to determine natural levels of rate variation relative to the performance of the different models.

**Results:**

Strict clock analyses performed well on data simulated under the independent rates model when the standard deviation of log rate on branches, σ, was low (≤0.1), but were inappropriate when σ>0.1 (95% of rates fall within 0.0082-0.0121 subs/site/Ma when σ = 0.1, for a mean rate of 0.01). The independent rates relaxed clock model performed well at all levels of rate variation, although posterior intervals on times were significantly wider than for the strict clock. The strict clock is therefore superior when rate variation is low. The performance of a correlated rates relaxed clock model was similar to the strict clock. Increased numbers of independent loci led to slightly narrower posteriors under the relaxed clock while older root ages provided proportionately narrower posteriors. The LRT had low power for σ = 0.01-0.1, but high power for σ = 0.5-2.0. Posterior means of σ^2 ^were useful for assessing rate variation in published datasets. Estimates of natural levels of rate variation ranged from 0.05-3.38 for different partitions. Differences in divergence times between relaxed and strict clock analyses were greater in two datasets with higher σ^2 ^for one or more partitions, supporting the simulation results.

**Conclusions:**

The strict clock can be superior for trees with shallow roots because of low levels of rate variation between branches. The LRT allows robust assessment of suitability of the clock model as does examination of posteriors on σ^2^.

## Background

Dating divergences between populations/taxa is of considerable value in phylogenetic/phylogeographic studies because of the importance of an absolute time-scale when assessing hypotheses of lineage diversification, e.g., [1-5]. Bayesian Markov Chain Monte Carlo (MCMC) methods have become widely used for this purpose [6-8]. The Bayesian approach is well-suited to dating because it naturally incorporates different sources of information and associated uncertainties through the priors. Most significantly, time calibrations are incorporated through the prior on divergence times. This represents a more robust solution to that offered by current maximum likelihood alternatives, which do not appear to correctly account for all sources of uncertainty and therefore underestimate confidence interval widths on divergence times [[9,10] pp248-251].

Bayesian MCMC dating incorporates models that allow the rate of molecular evolution to vary across the tree, through the prior on substitution rates. To date, rate variation is generally modelled using a relaxed or local clock approach in which the rate on a branch is either correlated with the rate on its ancestral branch or is independent of rates on other branches [11-13]. (Note that we use the term "relaxed clock" throughout this paper to simultaneously refer to both independent and correlated rates models.) Under the independent rates model, a rate is assigned to each branch from a single lognormal distribution. The program MCMCTREE [14] assigns both the mean rate and the variance of the log transformed rate, σ^2^, from gamma distributions specified by the user. This model is also implemented in the program BEAST [15] although the standard deviation of log rate, rather than σ^2^, is assigned from a user-defined distribution. Under the correlated rates model, rates on branches are dependent on branch durations and the rate on the ancestral branch. The mean of the normal distribution for log rate is obtained from the log of the rate on the ancestral branch. The variance of this distribution is the product of the branch time duration and a parameter ν that is specified from a gamma distribution [12]. Hence, rates on shorter branches will show greater similarity with the rate on their ancestral branches than rates on longer branches. Finally, strict clock models generally assign a single rate to the entire tree from a lognormal distribution with a mean and variance specified from a gamma or other distribution.

A comparison of the models discussed here indicated that the independent rates model performed well overall for simulated data, possibly because it can accommodate homogenous and correlated rates [7]. Nevertheless, this model contains more parameters and provides wider posterior intervals than the strict clock. Furthermore, the strict clock has been shown to perform well on data that show quite clock-like evolution [7]. An investigation of the impact of increasing levels of rate variation on the performance of the strict clock is therefore of considerable practical use. The correlated rates model has a large number of parameters but is also more restrictive than the independent rates model. It may therefore be a less suitable option than the other two models, under most conditions. Ho et al. [16] found that exponential and lognormal independent rates models performed well when rates were correlated or uncorrelated, but found little support for the correlated rates model (see also [17]). In contrast, Lepage et al. [18] compared the fit of several different models to three real datasets and argued for correlated rates particularly in large datasets (although their analyses did not use any calibrations and so did not fully reflect typical applications). Here, our primary aim was to compare independent rates with strict clock models, but we also examine the performance of the correlated rates model. We achieve this by analyzing divergence times of sequences using a strict phylogenetic approach, as opposed to divergence times of species using a phylogenetic-coalescent approach [19]. Also, we use programs that were designed for dating single topologies, rather than programs such as BEAST [15], because integration over topological uncertainty can have undesirable effects on the specification of priors on times [20]. Our approach attempts to simplify the analysis while still providing general findings concerning the suitability of the clock models and natural levels of rate variation.

This paper considers dating of shallow trees, which we broadly define as phylogenies with a Miocene or more recent root. There is good reason to believe that rates should be similar among recently diverged lineages. Rates may vary due to both stochastic effects and inherited or other lineage-correlated factors such as environment. Among the inherited effects, body size [21-25], mass-specific metabolic rate [26], but see also [27] and generation time [28-30] have all been suggested as partial explanations of rate variation. The similarity of these characteristics in closely related species leads to the expectation of lower rate variation in these phylogenies, which may favour use of a strict clock.

The decision to use a strict or relaxed clock needs to be informed by a suitable test. Although new methods are being developed [31], the likelihood ratio test (LRT) has traditionally been used for testing for clock-like evolution [32]. It compares a tree with no branch rate constraints with the same tree in which rates on branches are constrained to be equal. The LRT is powerful when rates vary between, but not within, branches [33] as modelled in the relaxed clock analysis. One disadvantage is that it may have low power when there are few taxa and sequences are short, leading to type II errors (incorrect acceptance of the clock). In addition, it will not detect rate variation if tips are all equidistant from the root, which could occur for example if equivalent rate changes occurred synchronously across all branches. There appears to be a paucity of detailed studies that consider the performance of the LRT. Hence, a secondary aim of this work was to consider its performance across different levels of rate variation.

This paper reports on the effects of rate variation on the recovery of node ages using strict and relaxed clock approaches as well as the ability of the LRT to detect this rate variation.

## Results

### Simulated data

#### 1) Rate heterogeneity

**Figure 1:**
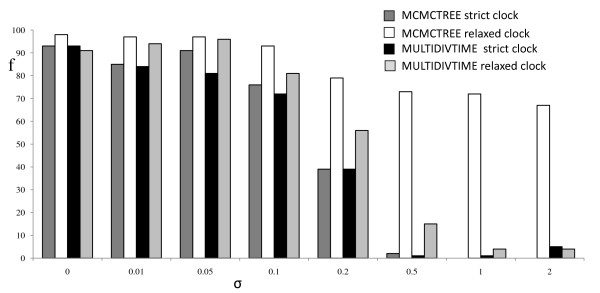
**Summaries of dating analyses on simulated data (frequencies)**. Frequencies of recovery of all node ages by strict and relaxed clock analyses in MCMCTREE and MULTIDIVTIME, for different standard deviations of the log rate (σ).

**Figure 2:**
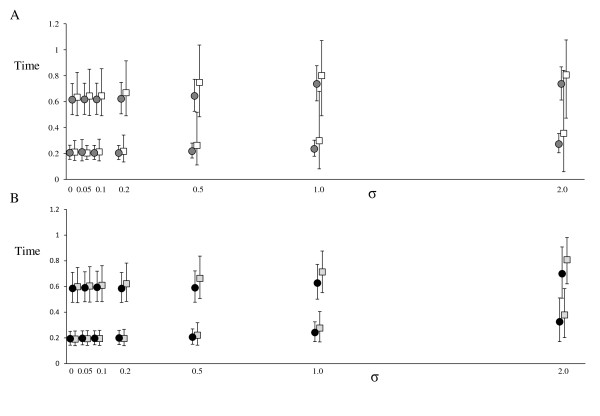
**Summaries of dating analyses on simulated data (posterior means)**. A. Posterior means and 95% interval widths (means from 100 simulations) for MCMCTREE strict clock (circles) and independent rates (squares) analyses for nodes with true ages of 0.6 and 0.2 (shown in Figure 4A). B. Posterior means and 95% interval widths (means from 100 simulations) for MULTIDIVTIME strict clock (circles) and correlated rates (squares) analyses.

The relaxed and strict clock analyses recovered all internal node ages on the tree in the majority of analyses when the sequences were evolved with σ ≤ 0.1 (Figure 1). Strict clock analyses performed poorly when σ > 0.1. Note that we use the term 'coverage probability' to describe the proportion of analyses that recover all node ages on the tree (see Methods). Coverage probabilities for relaxed clock analyses were high for all levels of σ under the independent rates model (MCMCTREE), but were significantly lower under the correlated rates model (MULTIDIVTIME) when σ>0.2. Posterior intervals on selected nodes were wider for relaxed clock analyses, markedly so when σ was high (Figure 2A, B). This effect was more noticeable under the independent rates than the correlated rates model. In contrast, the intervals remained similarly narrow for all levels of rate variation when analysed using a strict clock in MCMCTREE (Figure 2A).

MCMCTREE analyses of replicates simulated under σ = 2 that used the correlated rates instead of the independent rates model provided very similar results to the correlated rates MULTIDIVTIME analyses. Node ages were recovered for all nodes on the tree in only 4% of analyses, compared with recovery by 5% of analyses in MULTIDIVTIME. This contrasts with a recovery of all nodes in 67% of analyses under the independent rates model in MCMCTREE (Figure 1).

**Figure 3:**
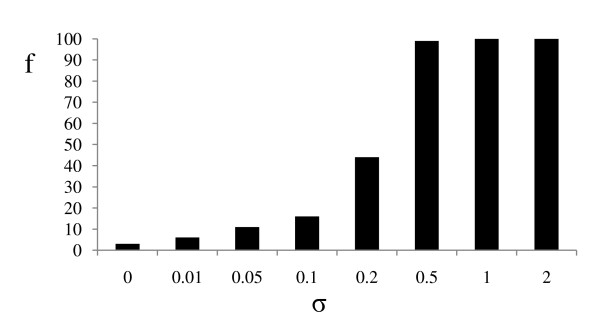
**Summary of likelihood ratio tests on simulated data**. Frequency of rejection of the clock for replicates generated under different σ.

The frequency of rejection of the clock by the LRT showed a sharp transition around σ = 0.1-0.2, which paralleled the performance of the strict clock analyses. The clock was rejected for less than 10% of the datasets evolved with σ < 0.2, but was almost always rejected when σ > 0.2 (Figure 3).

#### 2) Number of taxa

The independent rates analyses recovered all node ages in the 5 species tree for all replicates. Recovery of all nodes ages in the 10 species (97-100%) and the 20 species replicates (96-100%) was also high. The corresponding recovery of node ages under the correlated rates model was generally lower (89-99% for 5 species and 93-100% for 10 and 20 species trees). Slightly lower recovery of node ages was obtained under the strict clock, as would be expected given the previous results. The LRT rejected the clock for 11% (5 taxa), 16% (10 taxa) and 26% (20 taxa) of replicates.

#### 3) Age of root

The performance of the strict clock implemented in MULTIDIVTIME was strongly dependent on root age, with lower recovery of all node ages for older roots (48% of replicates for 20 Ma root (Ma is an abbreviation of Mega-annum) and 29% of replicates for 40 Ma root). There was slightly poorer recovery of all node ages under the correlated rates model at the oldest root age (69% of replicates) compared with trees with 5-20 Ma roots (81-83% of replicates). The pattern was different in MCMCTREE. Strict clock analyses seemed to perform slightly better when the root age was older (5 Ma, all node ages recovered in 68% of replicates; 10 Ma, 74%; 20 Ma, 85%; 40 Ma, 84%). The relaxed clock independent rates model performed more consistently over the four root ages (93-97% replicates). However, coverage probabilities were generally higher under relaxed compared with strict clocks over all root ages, for both programs. Posterior intervals on nodes became narrower, as a proportion of node age, with increasing root age under all relaxed clock analyses, but this trend was clearer in MCMCTREE. The LRT rejected the clock on slightly fewer occasions for 5 Ma (19%) and 10 Ma (16%) root ages, than for 20 Ma (22%) and 40 Ma (21%) root ages.

#### 4) Number of loci

Increasing the number of loci had no strong effect on the suitability of strict or relaxed clock analyses. For MULTIDIVTIME, recovery of all node ages was slightly higher for five independent loci under both the strict clock (80% of replicates) and correlated rates (85%), when compared with fewer loci (correlated rates: 77-81%, strict clock: 70-72%). Posterior interval widths were similar for different numbers of loci. Results differed for MCMCTREE, where an increased number of loci had no discernible effect on the frequencies of analyses recovering all node ages. There was a trend for narrower posteriors with increased numbers of loci for the MCMCTREE relaxed clock analyses but not for the strict clock analyses.

### Real datasets

#### 1) Black bass

The LRT indicated violation of the clock for codon position 2 (*X*^2 ^= 33.95, *P *= 0.03), but not for codon positions 1 (*X*^2 ^= 27.44, *P *= 0.12) or 3 (*X*^2 ^= 22.92, *P *= 0.29). The MCMCTREE independent rates analysis provided 95% posterior intervals for the variance in log rate, σ^2^, that included zero for codon position 1 (0.000, 0.358). The intervals were slightly higher for codon position 3 (0.077, 1.196), and indicated considerable rate variation at codon position 2 (0.209, 2.330). The posterior means for σ^2 ^corresponded to quite high values of σ ranging from 0.27-1.00.

Posterior node ages differed considerably between the strict and relaxed clock analyses with 95% intervals being wider and means lower under the relaxed clock for both programs (Table 1). MULTIDIVTIME posterior means were lower under the strict clock and higher under the correlated rates model, relative to the strict and independent rates models in MCMCTREE.

**Table 1:** MCMCTREE and MULTIDIVTIME strict and relaxed clock analyses of real datasets.

	MCMCTREE		MULTIDIVTIME	
	
	Strict Clock	Relaxed Clock	Strict Clock	Relaxed Clock
1) Black Bass				
Node 1	6.74 (5.67, 7.88)	7.85 (5.57, 10.71)	5.84 (4.85, 7.00)	9.33(6.54,12.84)
Node 2	2.62 (2.06, 3.25)	3.65 (2.14, 5.41)	2.06 (1.53, 2.64)	4.88 (2.67, 7.50)
2) Primate-sucking Lice				
Node 1	1.96 (1.53,2.42)	2.65 (1.65, 4.04)	1.58 (1.12, 2.15)	1.45 (0.79, 2.45)
Node 2	0.71 (0.53,0.92)	0.85 (0.48,1.35)	0.34 (0.21, 0.50)	0.61 (0.27, 1.20)
3) *Podarcis*				
Node 1	2.95 (2.32, 3.66)	3.20 (2.27, 4.49)	2.15 (1.25, 3.43)	2.31 (1.31, 3.94)
Node 2	1.14 (0.81, 1.52)	1.20 (0.79, 1.75)	0.65 (0.16, 1.32)	0.73 (0.06, 1.59)
4) Gallotiinae				
Node 1	5.87 (4.64, 7.16)	6.11 (4.67, 7.72)	5.84 (4.30, 7.48)	6.07(4.16, 8.11)
Node 2	1.71 (1.20, 2.30)	1.78 (1.15, 2.51)	1.58 (0.89, 2.33)	1.34 (0.61, 2.33)
5) Caprinae				
Node 1	6.01 (5.49, 6.78)	6.14 (5.41, 7.71)	5.69 (5.30, 6.91)	5.78 (5.31, 6.96)
Node 2	4.00 (3.21, 4.92)	4.48 (3.38, 5.87)	3.87 (2.82, 5.17)	4.47 (3.22, 5.95)

Posterior means and 95% intervals are given for nodes shown in Figs. 5A-E.

#### 2) Primate-sucking lice

The LRT did not indicate violation of the clock for mtDNA codon positions 1-3: *X*^2 ^= 4.29; *P *= 0.75; *X*^2 ^= 3.57, *P *= 0.83; *X*^2 ^= 7.16, *P *= 0.41, respectively, or for nuclear codon positions 1 or 2 (*X*^2 ^= 4.59, *P *= 0.71; *X*^2 ^= 1.34, *P *= 0.99, respectively). However, the clock was rejected for nuclear codon position 3 (*X*^2 ^= 26.25, *P *< 0.001) and the 18S rRNA gene (*X*^2 ^= 21.74, *P *< 0.003). The lower posterior limit on σ^2 ^(MCMCTREE independent rates analysis) approximated to zero for mitochondrial codon positions 1-3: (0.003, 0.840), (0.003, 0.794), (0.000, 1.987), and for nuclear codon positions 1 and 2: (0.003, 1.814), (0.000, 1.975). Evidence of higher rate variation was detected for nuclear codon position 3 (0.748, 4.247) and 18S rRNA (1.514, 6.182), closely reflecting the LRT results. The posterior means corresponded to a range of σ from 0.39-1.84.

For MCMCTREE, the ages of the two selected nodes differed between strict and independent rates analyses in the same way that they differed for the Black bass data, i.e., higher posterior means and wider posterior intervals for independent rates (Table 1). Posterior node ages were lower for both models in MULTIDIVTIME, relative to MCMCTREE.

#### 3) Podarcis

the LRT did not support violation of the clock for any of the partitions (cytochrome b, codon 1, *X*^2 ^= 11.40, *P *= 0.91; cytochrome b, codon 3, *X*^2 ^= 20.04, *P *= 0.39; ND1 and ND2 codon 1, *X*^2 ^= 14.43, *P *= 0.76; ND1 and ND2 codon 3, *X*^2 ^= 14.00, *P *= 0.78; 12S rRNA, *X*^2 ^= 2.92, *P *= 0.99; control region *X*^2 ^= 8.40, *P *= 0.98). The 95% posterior intervals on σ^2 ^also indicated low rate variation with lower posterior limits tending to zero in all cases: cytochrome b, codon 1, (0.001, 0.989); cytochrome b, codon 3, (0.001, 0.653); ND1 and ND2 codon 1, (0.005, 1.546); ND1 and ND2 codon 3, (0.002, 0.848); 12S rRNA, (0.002, 1.322); control region (0.002, 1.205), respectively. Posterior means were equivalent to a relatively narrow range of σ from 0.40-0.60.

Relaxed clock analyses again provided slightly wider posterior intervals on node ages than the strict clock (Table 1). Posterior means were lower under the strict clock compared with the relaxed clock but differences between these clock models were smaller than found in the Black bass and lice data. Again, posterior mean node ages were lower in MULTIDIVTIME than in MCMCTREE.

#### 4) The Gallotiinae

Similar to *Podarcis*, the LRT did not indicate violation of the clock for any of the mtDNA partitions (codon position 1, *X*^2 ^= 21.65, *P *= 0.42; codon position 2, *X*^2 ^= 12.69,*P = *0.92; codon position 3, *X*^2 ^= 21.47, *P = *0.43, rRNA loops *X*^2 ^= 20.96, *P = *0.46, rRNA stems *X*^2 ^= 17.04, *P = *0.71) which was consistent with the lower limits of the posteriors on σ^2^: codon position 1, (0.002, 0.448); codon position 2, (0.001, 0.901); codon position 3, (0.000, 0.215); rRNA loops (0.008, 1.088); rRNA stems (0.001, 0.541). Posterior means on σ^2 ^were quite low and equivalent to a relatively narrow range of σ from 0.25-0.57.

The posterior means showed generally the same patterns as those detected for *Podarcis*, i.e., relaxed and strict clock analyses gave quite similar mean node ages for both programs with slightly wider posterior intervals under the relaxed clock. Unlike *Podarcis*, there was no clear trend for the strict clock to always give lower posterior means than the relaxed clock or vice versa (Table 1).

We also explored the sensitivity of the posteriors to the prior on σ^2 ^using these data. The data were analysed with 5 different priors that ranged from the wide G(0.5, 0.01) to the unsuitably narrow G(0.5, 100)(Table 2). The three widest gamma distributions were all found to give similar results, indicating a considerable influence by the likelihood on the posterior for σ^2 ^in these cases.

**Table 2:** The impact of different priors on σ^2 ^in the Gallotiinae dataset (independent rates analysis).

Gamma prior	Codon 1	Codon 2	Codon 3	RNA stem	RNA loop
G(0.5, 0.01)	0.1089 (0.003, 0.563)	0.2695 (0.004, 1.358)	0.066 (0.002, 0.237)	0.444 (0.017, 1.532)	0.168 (0.002, 0.611)
G(0.5, 0.1)	0.116 (0.002, 0.583)	0.150 (0.000, 1.001)	0.060 (0.000, 0.224)	0.423 (0.014, 1.468)	0.173 (0.005, 0.619)
G(0.5, 1)	0.091 (0.002, 0.448)	0.193 (0.001, 0.901)	0.060 (0.001, 0.215)	0.319 (0.008, 1.088)	0.150 (0.001, 0.541)
G(0.5, 10)	0.023 (0.000, 0.142)	0.051 (0.001, 0.224)	0.040 (0.001, 0.144)	0.0833 (0.002, 0.323)	0.062 (0.000, 0.236)
G(0.5, 100)	0.0055 (0.000, 0.025)	0.004 (0.000, 0.023)	0.007 (0.000, 0.032)	0.006 (0.000, 0.028)	0.007 (0.000, 0.031)

Posterior means and 95% intervals (in parentheses) on σ^2 ^are shown for each data partition.

#### 5) Caprinae

The LRT indicated considerable violation of the clock at codon positions 1 and 3 (*X*^2 ^= 43.26, *P *< 0.01 and *X*^2 ^= 57.90, P < 0.001, respectively), but not at codon position 2 (*X*^2 ^= 31.24, *P = *0.12). Estimates of σ^2 ^do not reflect these results as clearly as for other datasets. The posterior intervals for σ^2 ^were quite low for all three codons: position 1, (0.012, 0.431); position 2, (0.003, 0.710); position 3 (0.056, 0.355). Posterior means were low with a narrow range, equivalent to σ = 0.35-0.42.

Posterior node ages from relaxed and strict clock analyses were quite similar for each program (Table 1). Differences between programs were also quite small for these data.

## Discussion

Analyses of our simulated data confirmed that the strict clock is useful for analysing shallow phylogenies. It provided relatively narrow posterior intervals and good recovery of node ages when rate variation between branches was low, that is, when the standard deviation of log rate on branches (σ) was ≤0.1. To better illustrate this: 95% of rates fall within the range 0.0082-0.0121 subs/site/Ma when σ = 0.01 (for a mean rate of 0.1 subs/site/Ma). The strict clock did not perform well when rate variation was higher. Relaxed clock analyses with independent rates showed a different performance profile. Coverage probabilities were similar or only slightly higher than the strict clock analysis when σ < 0.2 but were notably better when σ ≥ 0.2. At the highest level of rate variation (σ = 2), all internal node ages were recovered by 67% of analyses under the relaxed clock, compared with none under the strict clock. These results are partly explained by increased posterior intervals widths under the relaxed clock. The relaxed clock posteriors are substantially wider than corresponding strict clock intervals (44% wider when σ = 0.1). For this reason, the strict clock is preferable when rate variation is low but rapidly becomes unsuitable as rate variation increases.

Unlike the independent rates analyses, the correlated rates model did not perform well at higher levels of rate heterogeneity (σ = 0.2-2). This is attributed to the relaxed clock model rather than some other aspect of the programs because similarly poor performance was obtained when the correlated rates prior was tested in MCMCTREE. Our results strongly favour the independent over the correlated rates model when rate variation is high and not time-correlated between branches. However, correlated rates may be preferable to a strict clock at intermediate levels of rate variation.

We assessed performance in terms of coverage probabilities and posterior interval widths. These are the principal arbiters of a successful analysis because the aim is to achieve a high probability of capturing true ages within narrow posteriors. Accuracy of the posterior mean was not considered here, but it is worth noting that a small upward bias is evident in the posterior means of relaxed relative to strict clock analyses. This has been observed previously and seems to be associated with an increased influence by the prior on divergence times [34]. Why it might have greater influence at higher levels of rate variation will be explored in future work.

We demonstrate that robust assessment of the clock model is required prior to dating. The LRT is appropriate for this purpose when applied to the simulated data. It tended not to reject the clock when the strict model performed well (i.e., when σ < 0.2) and almost invariably rejected the clock when the strict clock model performed poorly (σ > 0.2). This finding changes a little for different numbers of species. The coverage probability averaged over all nodes remains quite similar for 5-20 species trees. However, the clock rejection rate by the LRT, shows more than a two-fold increase (from 11% of 5 species replicates to 26% of 20 species replicates) indicating that the clock is more likely be rejected for larger trees, even when the strict clock model is appropriate. More species on the tree increases the degrees of freedom in the LRT and increases statistical power. Hence violation from the clock is more likely to be detected even when rate variation is so small that the strict clock model is quite suitable. In contrast, the number of species on the tree should have no noticeable influence on σ.

An increased number of loci leads to a small improvement in performance. This result is specific to phylogenetic dating of sequences but not to phylogenetic-coalescent dating of speciation times, e.g., [19]. A small improvement could be expected in a phylogenetic analysis because a 'rate-outlier' on a branch will tend to have a significant impact when only one single locus is included. In a multilocus analysis the impact of such an outlier will be mitigated by rates at other independent loci on the same branch. These analyses also suggest that a strict clock may be suitable at slightly higher levels of rate variation when multiple independent loci are available.

From the simulations we expected that strict and relaxed clocks should perform similarly well when the LRT does not reject the clock. This is largely supported by the analyses of real datasets. Relaxed and strict clock analyses provided quite similar divergence time estimates for the Gallotiinae and Balearic *Podarcis *datasets, where rate variation was low for all data partitions. The clock was rejected for some sequence partitions in the other 3 datasets. Relaxed and strict clock analyses provided different divergence time estimates for two of these datasets, the Black bass and lice data, in which two or more partitions showed significant rate variation. The Caprinae data were an exception to these findings. The LRT rejected the clock for 2 out of 3 equally-sized partitions. In contrast, Bayesian estimates of σ^2 ^indicated relatively little rate variation which is consistent with the similarity of the strict and relaxed clock estimates of divergence times. This demonstrates the utility of the marginal posterior on σ^2 ^as a measure of rate variation across the tree. Unlike the variances in rates specified by the correlated rates model, which depend on branch duration, σ^2 ^provides a simpler estimate of rate variation. It could be more generally applied to compare across datasets. In BEAST [15] this could be achieved by examining the posterior on the standard deviation in log rate. Variance/standard deviation in rate will of course depend on mean substitution rate at the locus, and so comparisons would assume similar rates between loci.

Rate variation seems to vary quite widely between different genes and partitions in real datasets. Posterior means were equivalent to a range of σ from 0.2-1.8, which spans the range of σ examined in the simulations. Partitioning the data allows not only small improvements in divergence time estimation, as described here, but also detection of quite considerable differences in rate variation that can occur between partitions. Our analyses of real data indicate that significant rate variation in one partition justifies use of the relaxed clock, even though our more general conclusion is that the strict clock can be superior for analyses of recently diverged sequences.

## Conclusions

The strict clock is shown to have significant advantages over relaxed clocks because it provides good recovery of node ages and narrow posterior intervals when rate variation is low. Rate variation in three out of five shallow (Miocene root) phylogenies was within the range of rate variation over which the strict clock model performed well, supporting the applicability of the simulation results. The LRT is a generally suitable way to test the suitability of the strict clock, although examination of posteriors on σ^2 ^may be more informative.

## Methods

### Simulated data

**Figure 4:**
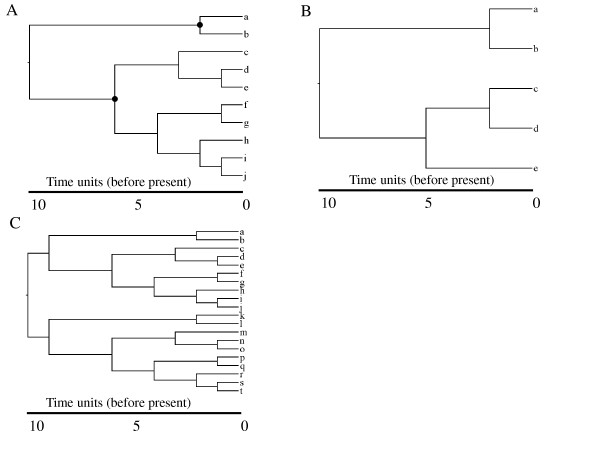
**Trees used in simulations**. The 10 species (A), 5 species (B), and 20 species (C) trees used in the simulations. Posteriors on nodes marked with filled circles are summarized in Figire 2.

Bayesian analyses are computationally intensive which favours simulation of smaller datasets. A tree with 10 taxa and a 10 Ma root (Figure 4A) was used for most simulations, although trees with different numbers of taxa (Figures 4B, C) and different node ages were also used. The general procedure was as follows. Rates were sampled from a log normal distribution with a mean of 0.01 substitutions/site/Ma and assigned to branches. Branch lengths were obtained from the product of branch duration and rate. DNA sequences of length 2500 bp were simulated for terminal taxa on these phylogenies using Evolver from the PAML suite of programs (ver. 4.3 [14]). The HKY+G model [35] was used with the following base frequencies:0.30 (T), 0.25 (C), 0.30 (A), 0.15 (G), a transition: transversion ratio (κ) of 5, and a gamma shape parameter (α) for site rate heterogeneity of 0.5. One hundred replicate datasets were generated for each set of conditions.

#### 1) Rate heterogeneity

Different levels of rate variation were simulated using the standard deviation of the normal distribution (σ) from which the natural logarithm of the rate was sampled. Replicate datasets were obtained for each of the following values of σ: 0 (strict clock), 0.05, 0.1, 0.2, 0.5, 1.0, 2.0. For illustration, the central 95% of rates will be between (0.0091-0.0110), (0.0066-0.0145) and (0.0000-0.0682) for σ = 0.05, 0.2 and 2.0, respectively.

#### 2) Number of taxa

Replicates were simulated for phylogenies containing 5, 10 and 20 species with σ = 0.1 (Figures 4B,C).

#### 3) Age of root

Replicates were simulated for the 10 species topology with the following root ages: 5 Ma, 10 Ma, 20 Ma, 40 Ma (σ = 0.1 in each case). The ages of other nodes on these trees were proportional to their ages on the tree with the 10 Ma root.

#### 4) Multiple loci

The 2500 bp of sequence was assigned to different numbers of partitions, each with a different rate but with σ = 0.1. The multiple loci tested were: two (1250 bp each, with mean rates 0.005 and 0.015), three (833 bp, rate = 0.005; 834 bp, rate = 0.010; 833 bp, rate = 0.015) and five (500 bp each, with mean rates 0.0050, 0.0075, 0.0100, 0.0125, and 0.0150) loci.

### Analyses of simulated data

Bayesian estimates of divergence times were obtained for each replicate using the programs MCMCTREE [14] and MULTIDIVTIME [36]. These programs were selected because their statistical features have been described and investigated quite extensively [6,8,12,33,36-39]. Details of the analyses are described below.

A multivariate normal approximation of maximum likelihood (ML) estimates of branch lengths is used by MULTIDIVTIME. Calculation of these branch lengths required assignment of an arbitrary outgroup to the simulated datasets. This was simulated in the same way as the other taxa, but with a divergence time that was 10% older than the root on each tree. (The outgroup was removed for the subsequent Bayesian dating analysis.) No outgroup was necessary for MCMCTREE, which calculates an exact likelihood using just ingroup sequences.

Analyses were specified to accommodate the models used to generate the simulated sequences. The correct (HKY) model was used with both i) κ (control variable: kappa_gamma) and ii) α (control variable: alpha_gamma) specified from the gamma distribution: G(1, 0.1). Gamma distributions are specified using the mean and standard deviation in MULTIDIVTIME, but for consistency we describe all gamma distributions in terms of shape (α) and scale (β) parameters where mean = α/β, variance = α/β^2^.

All analyses were carried out with 1 time unit = 10 Ma. Uniform distributions with hard bounds were used to constrain the root age in MCMCTREE (sensu [8]). This option was selected because soft bounds are not available in MULTIDIVTIME. The root was constrained by maximum (15% above the correct root age) and minimum bounds (15% below the correct root age).

The prior density on times in MCMCTREE, hereafter referred to as the BDS prior, was specified from a birth (λ), death (μ) process with species sampling (ρ), where λ = 5, μ = 5, ρ = 0.1. This specification generates higher densities of younger nodes for the range of true root ages used here (0.5-4 time units) and appears generally appropriate for analyses of shallow phylogenies [39]. In MULTIDIVTIME, the ages of the internal nodes on the tree are specified by a symmetric (i.e., all elements of the alpha vector are equal) Dirichlet distribution, conditional on the root age. The Dirichlet prior was parameterized by a single value defined by the minab option. We used minab = 1.0 which provides a uniform Dirichlet density. Prior intervals on node ages were assessed by running the MCMC chains without data.

In MCMCTREE relaxed clock analyses of simulated data, the hyperpriors on mean rate and the variance in log rate were both assigned from a gamma distribution: G(0.5, 1.0). In MULTIDIVTIME the hyperparameter that determines the degree of rate correlation, ν, was specified from G(0.5, 1.0). The rate at the root was also specified from the same gamma distribution. In order to separate the effects of the different models from other differences between programs, the correlated rates option was also used in MCMCTREE to analyse 10 species datasets (100 replicates) simulated for a 10 Ma root, using the same specification that was used in MULTIDIVTIME.

A single rate is specified for strict clock analyses. A G(0.5, 1.0) distribution was used to specify both the mean of this global rate and the variance of log rate in MCMCTREE, and also to specify the global rate in MULTIDIVTIME.

Bayesian MCMC chains were run for 2.5 × 10^5 ^generations, with a sampling interval of 50, for both programs. Likelihoods were obtained for rooted (clock) and unrooted (unconstrained) trees for each simulated dataset (individual loci analysed separately) using BASEML (ver. 4, [14]), and the significance of the difference between them tested using the LRT.

High coverage probabilities for individual node ages indicate that the analyses have performed well. However, wider posteriors increase the coverage probability without providing useful information on times. Performance was therefore assessed in two ways for each group of replicates: 1) examination of the coverage probabilities over all nodes, to obtain the frequency of replicates in which all node ages were recovered by the analysis, 2) analysis of mean widths of posterior intervals on selected nodes.

### Real datasets

Five datasets were used to examine levels of rate variation in real phylogenies and compare the results of strict and relaxed clock analyses. The LRT was used to test for clock-like evolution in individual partitions, for all datasets (ingroup taxa only).

#### 1) Black bass

**Figure 5:**
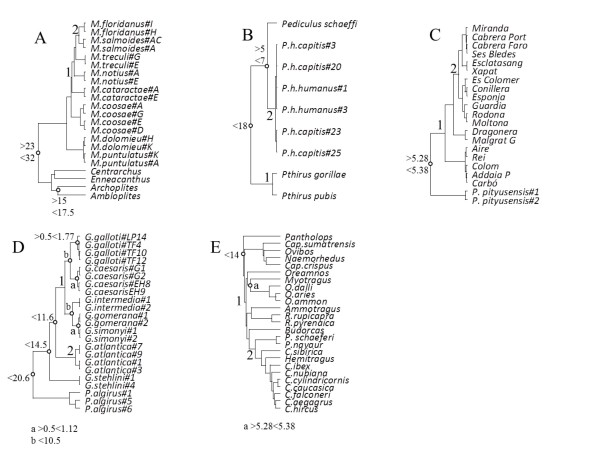
**Trees used for dating the five real datasets**. A. Black bass, B. Primate-sucking lice, C. *Podarcis *lizards, D. Gallotiinae, E. Caprinae. Trees are shown as chronograms which use the posterior mean node ages from the MCMCTREE relaxed clock analyses.

Phylogenetic relationships and divergence times among Black bass (*Micropterus*) were investigated by Near et al. [40] using 2190 bp of mtDNA, representing complete cytochrome b and ND2 gene sequences (Figure 5A, Additional file 1). Several additional genera were included (*Lepomis*, *Ambloplites*, *Archoplites*, *Ennacanthus*, *Centrarchus*) to provide minimum fossil constraints for two nodes. Minimum constraints alone are inadequate for reliable dating and so we used Near et al.'s findings to define reasonable maximum bounds on these nodes. The age of the most recent common ancestor (MRCA) of *Archoplites *and *Ambloplites *were specified as '>1.5 <1.75' time units, while the root node was specified as '>2.3 <3.2' time units (Figure 5A). We used hard bounds and specified 1 time unit = 10 Ma for all analyses of real data. *Lepomis *was used only as an outgroup for estimation of branch lengths, prior to the MULTIDIVTIME analyses. Similar sequences were removed, leaving 22 ingroup taxa for analysis.

Strict and relaxed clock analyses were performed using MULTIDIVTIME and MCMCTREE. Analyses were similar to those described for the simulated data, with minor differences which we describe here. In MCMCTREE, κ was specified from a G(5, 0.667) distribution and α was specified from a G(1, 1) distribution. Similarly, the mean rate was specified from a G(0.1, 1) distribution and the variance in log rate was specified from a G(0.5, 1) distribution both for rates on branches in the relaxed clock analysis and the global rate in the strict clock analysis. As for all data sets, priors on divergence times were examined by running the MCMC chain without data and then an appropriate BDS prior on times was specified (see [39]). For the Black Bass data this prior was specified as were: λ = 5, μ = 5, ρ = 1.

In MULTIDIVTIME the rate at the root (relaxed clock) and the global rate (strict clock) were specified from a G(0.1, 0. 1) distribution. The parameter, ν, was assigned from a G(0.5, 1) distribution. The Dirichlet prior on times was specified using minab = 0.3 to provide wide prior intervals on node ages.

#### 2) Primate-sucking lice

Speciation times in lice were investigated by Light and Reed [41], using sequences from COI, Cytochrome b, and CO3 mitochondrial genes and from 18SrRNA, EF-1a, CAD, POl, Wg, and H3 nuclear genes (Figure 5B, Additional file 2). We divided the sequence into 7 partitions: 3 partitions of 694 bp each corresponding to mtDNA codon positions, 3 partitions of 687 bp each corresponding to nuclear DNA codon positions, and 536 bp of nuclear sequence from the 18S rRNA. Some individuals originating from the most basal nodes were removed from the original dataset, leaving 9 individuals from the following species: *Pediculus schaeffi, P. humanus*, *Pthirus pubis *and *Pthirus gorilla *(Figure 5B). A tenth species, *Pedicinus badii*, was used as an outgroup in the MULTIDIVTIME analyses. All partitions were available for all ten species.

Light and Reed [41] applied 5-7 Ma constraints on the node representing the MRCA of *P. schaeffi *(chimpanzee lice) and *P. humanus *(human lice), corresponding to the divergence of humans and chimpanzees. We specified this calibration as well as a maximum bound on the root as of 18 Ma using the RootAge (MCMCTREE) and Bigtime (MULTIDIVTIME) control variables. The maximum bound on the root was specified using the posterior distribution published in the original study.

In MCMCTREE we specified the BDS prior on times as: λ = 5, μ = 5, ρ = 3. In MULTIDIVTIME the prior root age was specified from a G(4, 2.667) distribution. Other control variables were the same as those described for the Black bass analyses.

#### 3) *Podarcis *lizards

Brown et al. [34] analysed mtDNA from 21 *P. pityusensis *and *P. lilfordi *from different islands within the Balearics (Mediterranean sea) together with a *P. sicula *outgroup (used here to estimate branch lengths for MULTIDIVTIME only)(Figure 5C, Additional file 3). The sequence (control region, cytochrome b, ND1, ND2, 12S rRNA, 3 tRNAs) was divided into six partitions: cytochrome b codon position 1 (271 bp) and codon position 3 (270 bp), ND1/ND2 codon position 1 (149 bp) and codon position 3 (149 bp), control region (481 bp), 12S loops (213 bp). Other parts of the sequence (e.g., codon position 2) were removed because they contained negligible phylogenetic information.

The root node in the tree represents the MRCA of the two recognized species of Balearic *Podarcis*. Speciation coincided with the rapid refilling of the Mediterranean basin 5.33 Ma. Hence, Brown et al. [34] placed narrow 5.32-5.33 Ma constraints on the root node. We allowed slightly greater uncertainty here by specifying '>0.528 <0.538' (time units) as minimum and maximum constraints on the root. Other control variables were the same as for the Black Bass analyses, although analyses of priors on times indicated that a λ = 5, μ = 5, ρ = 3 BDS prior was more suitable in MCMCTREE.

#### 4) Gallotiinae lizards

Cox et al [42] analysed 1786 bp mtDNA from 20 *Gallotia *lizards endemic to the Canary archipelago, and three individuals from the North African sister taxon, *Psammodromus*. The sequence comprised 715 bp from the cytochrome b, 261 bp from cytochrome oxidase I, 414 bp from the 16s rRNA and 396 bp from the 12S rRNA genes (Figure 5D, Additional file 4). The lizard *Timon lepidus *was used as an outgroup in MULTIDIVTIME. Data were partitioned by codon position (cytochrome b and cytochrome oxidase I) and by stem and loop secondary structures (12S and 16S rRNA).

Maximum constraints on eight node ages were determined from island ages. These ranged from <2.06 time units on the root, to <0.112 time units on nodes that represent colonization of the youngest Canary Island, El Hierro. Arbitrary minimum constraints of >0.05 time units were also placed on the two (La Gomera, El Hierro) and the (Tenerife, La Palma) nodes, as in [42] (Figure 5D). In MULTIDIVTIME, the prior on age of the root was specified from a G(16, 8) distribution.

#### 5) Caprinae

Lalueza-Fox et al. [43] examined mtDNA evolution within the Caprinae. Brown and Yang [39] used an updated version of this dataset to obtain Bayesian estimates of divergence times in 25 Caprinae (Figure 5E, Additional file 5). We re-examined the dataset used in [39]. The data consist of 1128 bp of the cytochrome b gene, after removal of missing data, and were partitioned by codon position (Figure 5E). Twenty-five species were analysed, together with an outgroup for MULTIDIVTIME.

Constraints were applied to two different nodes (Figure 5E). First, the constraint '>0.528 <0.538' was placed on the (*Myotragus*, *Ovis*) node. This corresponds to the same physical event that was described for Balearic *Podarcis*. Second, we added a '<1.4' maximum bound on the root, which Lalueza-Fox et al. [43] reported as the earliest likely time for the origin of this radiation. This was specified using the RootAge control variable in MCMCTREE and the Bigtime variable in MULTIDIVTIME. In MULTIDIVTIME the root age was specified from a G(4, 6.4516) distribution.

## Authors' contributions

RPB carried out the analyses and drafted the manuscript. ZY participated in its design and helped draft the manuscript. Both authors read and approved the final manuscript.

## Supplementary Material

Additional file 1 **Black bass data**. Sequences are in Phylip format. Partitions are in the order described in the manuscript. Click here for file

Additional file 2 **Primate-sucking Lice data**. Sequences are in Phylip format. Partitions are in the order described in the manuscript. Click here for file

Additional file 3 ***Podarcis *data**. Sequences are in Phylip format. Partitions are in the order described in the manuscript. Click here for file

Additional file 4 **Gallotiinae data**. Sequences are in Phylip format. Partitions are in the order described in the manuscript. Click here for file

Additional file 5 **Caprinae data**. Sequences are in Phylip format. Partitions are in the order described in the manuscript. Click here for file
